# Genetic Determinants of Premature Menopause in A Mashhad Population Cohort

**DOI:** 10.22074/IJFS.2020.134688

**Published:** 2021-01-27

**Authors:** Mohammad Reza Mirinezhad, Narges Khosroabadi, Maliheh Rahpeyma, Reza Khayami, Seyyed Reza Hashemi, Hamideh Ghazizadeh, Gordon A Ferns, Alireza Pasdar, Majid Ghayour-Mobarhan, Tayebeh Hamzehloei

**Affiliations:** 1. Department of Medical Genetics and Molecular Medicine, Faculty of Medicine, Mashhad University of Medical Sciences, Mashhad, Iran; 2. Department of Genetics, Faculty of Biological Science, Shahid Beheshti University, Tehran, Iran; 3. Student Research Committee, Mashhad University of Medical Sciences, Mashhad, Iran; 4. Metabolic Syndrome Research Center, Mashhad University of Medical Sciences, Mashhad, Iran; 5. International UNESCO Center for Health-Related Basic Sciences and Human Nutrition, Mashhad University of Medical Sciences, Mashhad, Iran; 6. Brighton and Sussex Medical School, Division of Medical Education, Falmer, Brighton, Sussex BN1 9PH, UK; 7. Division of Applied Medicine, Medical School, University of Aberdeen, Foresterhill, Aberdeen, AB25 2ZD, UK

**Keywords:** Association Studies, Genetic Polymorphisms, Haplotype, Premature Menopause

## Abstract

**Background:**

Premature menopause is characterized by amenorrhea before age of 40 years, markedly raised serum luteinizing hormone (LH) level, follicle-stimulating hormone (FSH) level and reduced serum level of estradiol. Genome-wide analysis suggested several loci associated with premature menopause. Here, we aimed to analyze association of variants at the *MCM8, FNDC4, PRRC2A, TLK1, ZNF346* and *TMEM150B* gene loci with premature menopause.

**Materials and Methods:**

In this cross-sectional study, a total of 117 women with premature menopause were compared to 183 healthy women. Anthropometric indices were measured in all participants: height, weight, body mass index (BMI), waist circumference (WC) and wrist circumference. Eight single-nucleotide polymorphisms (SNPs) of the indicated genes (rs16991615, rs244715, rs451417, rs1046089, rs7246479, rs4806660, rs10183486 and rs2303369) were identified from the literature. Genotyping was performed using tetra-ARMS polymerase chain reaction (PCR) and ASO-PCR methods.

**Results:**

T allele of the rs16991615, rs1046089, rs7246479 and rs10183486, C allele of rs244715, rs451417 and rs4806660 as well as TT genotype of rs2303369 were associated with an increased risk of premature menopause, likely causing susceptibility to primary ovarian insufficiency (POI) in comparison with C allele. We also found an association between the rs16991615 SNP with premature menopause. Frequency of the minor allele in cases was increased for all SNPs in comparison with controls. All minor alleles, except for rs2303369, showed a statistically significant increased odds ratio (OR). However, after Bonferroni correction for multiple testing, none of the P values were remained significant.

**Conclusion:**

The selected polymorphisms in *MCM8, FNDC4, PRRC2A, TLK1, ZNF346* and *TMEM150B* genes may potentially affect susceptibility to premature menopause, although replication of the results in larger cohort could clarify this.

## Introduction

Premature menopause or primary ovarian insufficiency (POI) is the occurrence of menopause before age of 40 years (1). This condition is characterized by amenorrhea, increased luteinizing hormone (LH) level, follicle-stimulating hormone (FSH) level and reduced level of estradiol. POI may be idiopathic or associated with autoimmune and genetic abnormalities (2). Other than that, factors such as infections, chemo and radiotherapy, surgery and adverse effects of drugs could also be contributory (3, 4). POI may lead to increase of all-cause mortality, cardiovascular diseases, depression, type 2 diabetes and increased risk of bone fracture (5-8). 

Genetic factors appear to be responsible for approximately 50% of the variations in the onset of menarche and menopause (9). Several studies have found different genetic loci associated with age at natural menopause (ANM) (10-14). DNA repair genes such as *EXO1, HELQ, UIMC1, FAM175A, FANCI, TLK1, POLG* and *PRIM1* along with genes like *IL11, NLRP11* and *PRRC2A* which are involved in immune function, are located at these loci (14). 

Mutations in some of these genes have been indicated to be involved in POI. For example, Tenenbaum-Rakover et al. (15) found two new homozygous mutations in the *MCM8* gene, a frameshift (c.1469-1470insTA) and a splice site mutation (c.1954-1G>A). These mutations were shown to increase the risk of chromosomal breakage after mitomycin C exposure. The authors also found that *MCM8* plays a pivotal role in gonadal development. Desai et al. (16) also found that mutations in *MCM8* and *MCM9* could be a significant risk factor for POI. Besides, several other studies have replicated findings obtained from rs16991615 polymorphism, located in *MCM8*, as a significant single-nucleotide polymorphism (SNP) associated with ANM in different populations (17, 18). Llácer et al. (19) showed that *IL11*- rs11668344 and *PRRC2A*rs1046089 are associated with poor ovarian response indicating that women carrying these polymorphisms have decreased oocyte production in response to controlled ovarian hyper stimulation. On this basis, we selected eight different SNPs in the genes more commonly associated with POI. They have not previously been studied in the Iranian population. We aimed to determine whether these SNPs are involved in the development of premature menopause and to find their association with anthropometric characteristics in a population sample from Mashhad city in Iran.

## Materials and Methods

### Studying participants and anthropometric indices

In this cross-sectional study, a total of 117 women who were originally enrolled in the Mashhad stroke and heart atherosclerotic disorder (MASHAD) study were recruited and compared to 183 healthy women. "MASHAD study" is a 10-years cohort study of a total of 9704 individuals aged 35-65 years, from an urban population in eastern part of Iran. They were selected using a stratified cluster random sampling design. The healthy group was chosen from women without history of menopause before the age of 40 years to match with the cases group in terms of age. The cases were also selected from this study cohort based on their history of premature menopause. A complete history was taken for all subjects, including any surgical procedures, acute or chronic diseases, chemotherapy or radiation therapy, smoking habit, medications as well as the results of periodic examinations. Women who were not willing to enroll in the present study, were excluded. We included all eligible and available volunteer women who met the study criteria. To clarify this, 9704 people participated in "MASHAD study" project. 5838 of the cases were women. 2747 women reached menopause by the time of starting study. Furthermore, 895 women were removed from the project (due to hysterectomy, oophorectomy and other secondary causes). Out of the 1852 remaining women, 117 (all the cases existed in the population) cases were below the age of 40 years at their menopause. They were selected according to the POI definition, including: i. Women going through menopause before the age of 40 years, ii. 12 months of consecutive menstrual cessation or iii. Elevated serum FSH levels >40 IU/L (repeated at four-week intervals) (1). Other inclusion criteria were provided as follows: women with POI, women younger than 40 years old, women without history of diseases affecting menstruation, women without any genetically confirmed diseases or syndromes that early menopause was part of their manifestations, women without any previous surgeries affecting menstruation (oophorectomy, hysterectomy) and women who were not using any drugs affecting menstruation. Hence, 117 women were considered eligible to be enrolled in the present study. Anthropometric indices such as height, weight, body mass index (BMI), waist circumference (WC) and wrist circumference were measured in all participants. Informed consent was obtained from all participants. The approval number from the constituted review board, the Ethics Committee of Mashhad University of Medical Sciences is IR.MUMS.MEDICAL.REC.1398.658.

### Genotyping

### DNA extraction and quality control

Total genomic DNA was extracted from 200 µl blood or buffy coat using a DNA extraction kit (Parstous, Iran). After DNA extraction, the samples were loaded and run on agarose gel (Parstous, Iran). Quantification of the DNA samples was evaluated by Nanodrop 2000 (Thermo Fisher Scientific, USA) at wave length of 280 and 260 nm.

### Tetra-ARMS polymerase chain reaction

Tetra-ARMS polymerase chain reaction (PCR) was carried out in 15 µl reaction volume containing 1 µl of each primer (0.6 pM of each primer), 7.5 µl master mix (Parstous, Iran), 2 µl genomic DNA and 1.5 µl water. Primers were designed using Primer 1 software. TetraARMS PCR was conducted with an initial denaturation step at 95°C for 5 minutes, followed by 30 cycles of denaturation at 94°C for 30 seconds, annealing at 58°C for 30 seconds and extension at 72°C for 40 seconds. Final extension step was performed at 72°C for 5 minutes. The sequences of all primers are summarized in Table S1 (See Supplementary Online Information at www.ijfs.ir). Sanger sequencing was performed to confirm the results.

### Allele-specific oligonucleotide polymerase chain reaction

**Fig.1:**
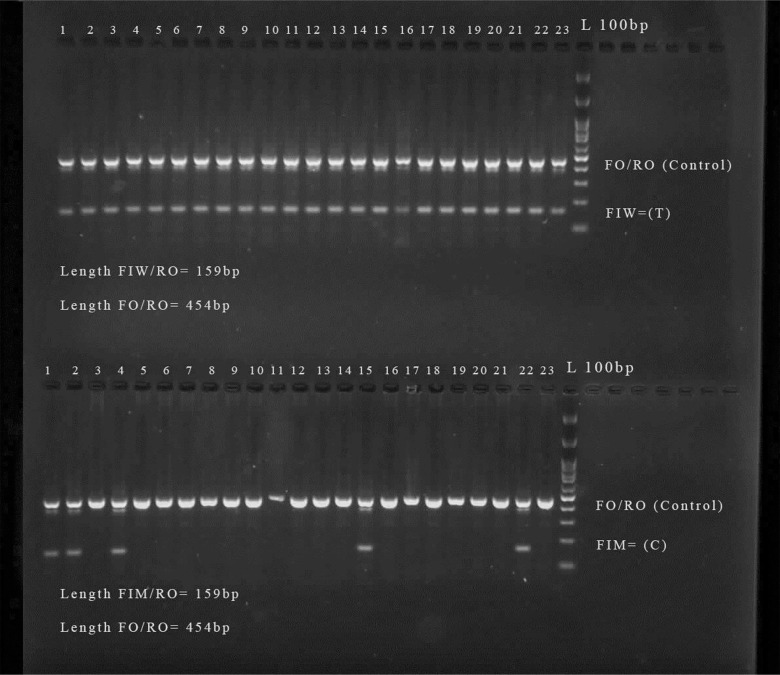
The RS4806660 Gel electrophoresis. Tetra- ARMS polymerase chain reaction (PCR) products were loaded and run on 2% agarose gel.

Allele-specific oligonucleotide PCR (ASO-PCR) was performed in a 15 µl reaction volume containing 1 µl of each primer (4 µM), 7.5 µl master mix (Parstous, Iran), 2 µl genomic DNA and 1.5 µl water. Primers were designed using Primer 3 software. PCR was carried out with the following condition: one cycle of initial denaturation at 95°C for 7 minutes, followed by 35 cycles including 95°C for 30 seconds, annealing for 30 seconds, 72°C for 30 seconds, followed by one cycle of 7 minutes for the final extension. The sequences of all primers are summarized in Table S1 (See Supplementary Online Information at www.ijfs.ir). The sample data are presented in Figure 1. 

### Statistical analysis

All experiments were performed in triplicate and reported values were displayed as mean ± standard deviation (SD). All data were analyzed using Microsoft Excel and SPSS version 24 (SPSS Inc., USA). t test statistical analysis was applied for comparing mean differences between two independent groups, and One-way ANOVA test was used to compare mean differences between more than two groups. Chi-square test was performed to analyze the Hardy-Weinberg equilibrium. Binary logistic regression test was used to calculate the odds ratio (OR) and confidence interval (CI) based on genotype data. Univariate and multivariate analysis were applied for the polymorphisms. Significance level is shown with *P<0.05, ** P<0.01 and ***P<0.001. Where applicable, Bonferroni correction was used to correct for multiple testing.

## Results

### Study of population characteristics

The mean age was 55.21 ± 5.56 years for the cases and 54.62 ± 2.89 years for the controls (P=0.411). A significant difference was observed between the average heights, waist circumference, weight, waist/height ratio and waist/hip ratio (WHR) of both groups. The controls were significantly heavier (70.96 ± 10.80 Kg) than the cases (67.11 ± 12.03 Kg, P<0.001). In contrast, the waist/height ratio was higher in the patients (63.55 ± 8.41) compared to the healthy subjects (60.30 ± 6.84, P=0.001). WHR was also greater in patients (0.93 ± 0.08) than controls (0.89 ± 0.07, P=0.001). The data are summarized in Table 1.

**Table 1:** Demographic features and characteristics of the study population


Indices	Case	Control	P value

Age (Y)	55.21 ± 5.56	54.62 ± 2.89	0.411
Height (m)	1.53 ± 0.06	1.56 ± 0.06	0.001
Waist circumference (cm)	96.96 ± 12.19	93.70 ± 10.44	0.020
Weight (kg)	67.11 ± 12.03	70.96 ± 10.80	<0.001
Hip circumference (cm)	104.71 ± 9.85	105.57 ± 8.78	0.430
Waist/height ratio	63.55 ± 8.41	60.30 ± 6.84	0.001
Waist/hip ratio	0.93 ± 0.08	0.89 ± 0.07	0.001
Mid upper circumference (cm)	30.69 ± 4.47	31.02 ± 4.61	0.550
Body mass index (kg/m^2^)	28.78 ± 5.06	29.34 ± 4.22	0.300


Data are presented as mean ± SD.

### Association of the single nucleotide polymorphisms and premature menopause

A total of eight SNPs were investigated in this study: rs16991615, rs244715, rs451417, rs1046089, rs7246479, rs4806660, rs10183486 and rs2303369. The genotype and allele frequencies of these SNPs are shown in Table 2. All SNPs conformed to the Hardy-Weinberg equilibrium. We found that the T allele of rs16991615, rs1046089, rs7246479 and rs10183486 as well as the C allele of rs244715, rs451417 and rs4806660 were associated with increased risk of premature menopause, in addition to the TT genotype of rs2303369 which may increase the susceptibility to POI in comparison with C allele careers. Besides, using a recessive model, genotypes of all SNPs were associated with an elevated risk of the disease (Table 3). Our results also suggested that CT genotype of rs7246479 was associated with increased susceptibility to premature menopause in comparison with CC genotype (OR=1.92, 95% CI=1.16-3.18, P=0.01). Furthermore, dominant model showed that genotype frequency of CC+TC was associated with increased risk of premature menopause for rs244715 (OR=1.70, 95% CI=1.06-2.72, P=0.027) in comparison with TT, while the T allele careers of rs1046089 (OR=1.71, 95% CI=1.05-2.79, P=0.03) and rs7246479 (OR=2.13, 95% CI=1.30-3.47, P=0.002) showed a greater association with risk for POI in comparison with CC. The additive model also indicated that TT genotype of rs16991615, rs1046089, rs10183486 and rs2303369, as well as CC genotype of rs451417 and rs4806660, were associated with increased risk of premature menopause, more than 100%. We acknowledge that although in the initial analysis some of these SNPs seemed to be associated with premature menopause, after correction for multiple testing none of them remained significant, as explained in Table 4.

**Table 2:** Genotype distribution and allele frequencies of the single nucleotide polymorphism (SNPs)


SNP	Genotype frequency										HWE
	Case (117)			Alleles		Control (183)			Alleles		P value
	AA	Aa	aa	A	a	AA	Aa	aa	A	a	

rs16991615 (G>A)	44	39	29	0.57	0.43	96	79	18	0.66	0.33	0.95
rs244715 (A>G)	59	46	12	0.70	0.3	116	61	6	0.80	0.20	0.83
rs451417 (A>C)	48	36	33	0.56	0.43	87	76	20	0.68	0.32	0.85
rs1046089 (G>A)	37	59	21	0.56	0.44	81	89	13	0.69	0.31	0.22
rs7246479 (G/T)	36	67	14	0.60	0.4	89	86	8	0.72	0.28	0.07
rs4806660 (T>C)	50	53	14	0.65	0.34	94	82	7	0.74	0.26	0.10
rs10183486 (C>T)	47	55	15	0.64	0.36	93	81	9	0.73	0.27	0.25
rs2303369 (C>T)	42	54	21	0.59	0.41	77	90	16	0.67	0.33	0.35


**Table 3:** OR based on different models


SNP	Additive model (aa vs. Aa)	P value	Dominant model ( aa+Aa vs. AA)	P value
	OR (95% CI)		OR (95% CI)	

rs16991615 (G>A)	3.26 (1.61-6.58)	0.001	1.52 (0.95- 2.45)	0.078
rs244715 (A>G)	2.65 (0.92-7.59)	0.069	1.70 (1.06-2.72)	0.027
rs451417 (A>C)	3.48 (1.76-6.89)	<0.001	1.30 (0.81-2.08)	0.269
rs1046089 (G>A)	2.43 (1.13-5.24)	0.023	1.71 (1.05-2.79)	0.03
rs7246479 (G/T)	2.24 (0.89-5.66)	0.087	2.13 (1.30-3.47)	0.002
rs4806660 (T>C)	3.09 (1.17-8.16)	0.023	1.41 (0.88-2.25)	0.145
rs10183486 (C>T)	2.45 (1.00-6.00)	0.050	1.53 (0.96-2.46)	0.070
rs2303369 (C>T)	2.18 (1.05-4.55)	0.040	1.29 (0.80-2.09)	0.290
	Allelic model (a vs. A)		Homozygote contrast (aa vs. AA)	
rs16991615 (G>A)	1.79 (1.27-2.53)	0.001	3.51 (1.76-6.99)	0.001
rs244715 (A>G)	1.71 (1.17- 2.50)	0.005	3.93 (1.40-11.00)	0.009
rs451417 (A>C)	1.66 (1.18-2.33)	0.003	2.99 (1.54-5.77)	0.001
rs1046089 (G>A)	1.65 (1.17-2.32)	0.004	3.53 (1.59-7.81)	0.002
rs7246479 (G/T)	1.76 (1.25-2.50)	0.001	4.32 (1.67-11.19)	0.003
rs4806660 (T>C)	1.48 (1.042-2.12)	0.028	3.76(1.42-9.91)	0.007
rs10183486 (C>T)	1.53 (1.08-2.18)	0.020	3.29 (1.34-8.09)	0.010
rs2303369 (C>T)	1.39 (0.99-1.95)	0.060	2.40 (1.13-5.10)	0.020
	Heterozygote contrast (Aa vs. AA)		Recessive model ( aa vs. Aa+AA)	
rs16991615 (G>A)	1.07 (0.63-1.81)	0.781	3.39 (1.78-6.46)	0.001
rs244715 (A>G)	1.48 (0.90-2.43)	0.119	3.37 (1.22-9.24)	0.018
rs451417 (A>C)	0.85 (0.50-1.45)	0.573	3.20 (1.73-5.92)	0.001
rs1046089 (G>A)	1.45 (0.87-2.41)	0.152	2.86 (1.37-5.96)	0.005
rs7246479 (G/T)	1.92 (1.16-3.18)	0.010	2.97 (1.20-7.32)	0.018
rs4806660 (T>C)	1.21 (0.74-1.97)	0.433	3.41 (1.33- 8.74)	0.010
rs10183486 (C>T)	1.34 (0.82-2.19)	0.240	2.84 (1.20- 6.73)	0.020
rs2303369 (C>T)	1.1(0.66-1.82)	0.710	2.28 (1.13-4.58)	0.020


SNP; Single nucleotide polymorphism, OR; Odds ratio, and CI; Confidence interval.

**Table 4:** Association between the SNPs and demographic characteristics


Polymorphism			Height (m)	Waist circumference (cm)			Weight (kg)		Hip circumference (cm)		Waist/ height ratio		Waist/hip ratio	Mid upper circumference (cm)		BMI (kg/m^2^)	

rs16991615																	
AA (n=134)		Mean	1.54604	95.5396			69.8507		105.3455		61.8738		0.9069	30.7632		29.2723	
		SD	0.059774	11.59249			11.74		9.636		7.79393		0.07493	4.06007		4.89012	
Aa+aa (n=166)		Mean	1.54366	94.4811			69.1659		105.1543		61.2964		0.8992	30.9902		29.0056	
		SD	0.058927	10.96122			11.19606		8.85304		7.52282		0.07828	4.93033		4.29641	
		P value^*^	0.730	0.420			0.608		0.859		0.517		0.386	0.670		0.617	
rs244715																	
AA (n=172)		Mean	1.54413	94.932			68.443		104.625		61.5679		0.9077	30.4241		28.722	
		SD	0.058418	11.08496			10.86428		8.78544		7.605		0.07768	2.99326		4.37843	
Aa+aa (n=128)		Mean	1.54556	94.9913			70.881		106.0802		61.5399		0.8958	31.5143		29.6764	
		SD	0.060525	11.49888			12.06058		9.70565		7.71405		0.07526	6.01161		4.77505	
		P value	0.838	0.964			0.069		0.178		0.975		0.186	0.042		0.075	
rs451417																	
AA (n=135)		Mean	1.5443	95.0711			68.9215		105.0119		61.6286		0.9056	30.6828		28.8994	
		SD	0.061006	11.20656			10.88902		8.65259		7.47528		0.07947	3.80482		4.27702	
Aa+aa (n=165)		Mean	1.54509	94.8626			69.9313		105.4294		61.496		0.9002	31.058		29.3128	
		SD	0.057888	11.30614			11.87232		9.64887		7.7933		0.0746	5.09652		4.79884	
		P value	0.908	0.874			0.449		0.697		0.882		0.549	0.481		0.438	
rs1046089																	
AA (n=108)		Mean	1.53991	94.9944			69.0157		105.1648		61.7733		0.9031	30.5252		29.1215	
		SD	0.055895	10.98759			11.2755		8.80033		7.57492		0.0696	3.06539		4.63562	
Aa+aa (n=192)		Mean	1.54747	94.9358			69.7342		105.2832		61.4326		0.9024	31.0937		29.1279	
		SD	0.061005	11.41384			11.53757		9.43923		7.69138		0.08073	5.20816		4.53979	
		P value	0.290	0.966			0.603		0.915		0.712		0.938	0.303		0.991	
rs7246479																	
AA (n=125)		Mean	1.53992	95.5864			70.3928		105.2984		62.132		0.9078	30.722		29.6871	
		SD	0.060728	10.95757			11.09858		9.25444		7.25764		0.06692	3.85928		4.40168	
Aa+aa (n=175)		Mean	1.54821	94.5023			68.8098		105.1983		61.1399		0.899	31.0064		28.7198	
		SD	0.058037	11.45425			11.64944		9.18376		7.89665		0.08315	4.99693		4.65319	
		P value	0.234	0.412			0.239		0.926		0.269		0.329	0.597		0.071	
rs4806660																	
AA (n=144)		Mean	1.54222	96.3625			67.6604		104.7625		62.5597		0.92	30.5895		28.4628	
		SD	0.056426	11.34757			11.4973		8.87954		7.68153		0.07821	3.12603		4.67753	
Aa+aa (n=156)		Mean	1.54708	93.6429			71.1695		105.687		60.6176		0.8864	31.1673		29.7452	
		SD	0.061813	11.0187			11.13753		9.4933		7.50157		0.07191	5.5638		4.38612	
		P value	0.480	0.037			0.008		0.387		0.028		<0.001	0.276		0.015	
rs10183486																	
AA (n=140)		Mean	1.55436	96.3093			71.2586		106.7629		62.054		0.9025	31.3266		29.491	
		SD	0.061752	10.84882			11.72958		9.3348		7.45174		0.07029	4.26855		4.52896	
Aa+aa (n=160)		Mean	1.5362	93.7589			67.8924		103.8911		61.1148		0.9028	30.5		28.8017	
		SD	0.055702	11.48176			10.95238		8.8882		7.79695		0.0823	4.77225		4.59034	
		P value	0.008	0.050			0.011		0.007		0.290		0.975	0.119		0.194	
rs2303369																	
AA (n=119)		Mean	1.54345			96.8336	67.5714		104.9563		62.8264		0.9232	30.7568		28.3835	
		SD	0.054779			11.18619	11.66716		8.84538		7.69302		0.08058	2.98854		4.79756	
Aa+aa (n=181)		Mean	1.54559			93.7095	70.7385		105.4291		60.7116		0.889	30.9753		29.6188	
		SD	0.062135			11.1368	11.12204		9.44499		7.50472		0.07112	5.35162		4.35084	
		P value	0.760			0.019	0.019		0.665		0.019		<0.001	0.687		0.022	


; Due to multiplecomparison, Bonferroni correction has been applied and the level of significance is set to <0.006 and SNP; Single-nucleotide polymorphism.

**Table 5:** Univariate multivariate analysis of the polymorphisms between case and control groups


SNP	Univariateanalysis	Multivariateanalysis

rs16991615	0.312	0.162
rs244715	0.008	0.019
rs451417	0.270	0.589
rs1046089	0.208	0.095
rs7246479	0.286	0.600
rs4806660	0.005	0.024
rs10183486	0.072	0.096
rs2303369	0.008	0.028
Age (Y)	0.163	
Height (m)	0.310	
Waist circumference (cm)	0.439	
Weight (kg)	0.830	
Hip circumference (cm)	0.938	
Waist: height ratio	0.550	
Waist:hip ratio	0.348	
Mid upper circumference (cm)	0.332	
Body mass index (kg/m^2^)	0.463	


Univariate and multivariate linear regression were used to indicate the polymorphisms correlations. * ; Due to multiple comparison, Bonferroni correction was applied and the level of significance was set to <0.003.

### Association of the single nucleotide polymorphisms with demographic characteristics

We found no significant association between rs16991615, rs451417 and rs1046089 polymorphisms and demographic characteristics of the patients. Additionally, only the rs244715 showed an association with Mid-Upper Arm Circumference (MUAC) in which the dominant homozygous genotype had lower MUAC (P=0.042). WC and weight did not seem to be associated with rs4806660, rs10183486 and rs2303369 (P=0.05). In all of these three SNPs, the dominant homozygous genotype had a greater WC. On the other hand, people with the dominant homozygous genotype of rs10183486 were heavier, while the same genotype for rs2303369 and rs4806660 had lower weight. Hip circumference only showed association with rs10183486, as dominant homozygous genotype. Furthermore, waist/height and waist/hip ratios were significantly higher for the dominant homozygous genotype of rs4806660 and rs2303369. Finally, women having TT and CC genotypes for the rs4806660 and rs2303369, respectively, had significantly higher BMI. The association of these SNPs and demographic characteristics are summarized in Table 4. However, after correcting for multiple testing using Bonferroni correction, they were not remained significant. Moreover, univariate regression analysis (model 1) was used to analyze the association between genetic variants and risk of POI. While, multivariate regression analysis (model 2) was adjusted for sex, age, smoking status, BMI, WC, waist/hip, waist/height.

## Discussion

We evaluated association of eight different SNPs including rs16991615, rs244715, rs451417, rs1046089, rs7246479, rs4806660, rs10183486 and rs2303369 with premature menopause. All of these SNPs except for rs244715, rs7246479 and rs4806660 were previously identified in GWAS. These SNPs are mapped to different loci in the genome. rs16991615 and rs451417 are mapped to *MCM8* while rs2303369, rs1046089, rs10183486 and rs244715 are mapped to *FNDC4, PRRC2A, TLK1* and *ZNF346*, respectively. Besides, rs7246479 and rs4806660 are both mapped to* TMEM150B*. 

Several studies have established the role of genetic variations in premature menopause. One of the loci is located on chromosome 19 containing MCM8, which encodes a mini-chromosome maintenance protein called DNA replication licensing factor MCM8. MCM8 plays role in DNA repair (16). Moreover, this protein has an important role in gametogenesis as *MCM8* knockedout mice showed sterility and arrested primary follicles in males and females, respectively (20). Additionally, *MCM8* expression was increased during the follicular phase of menstrual cycle in humans (21). Mutations in this gene have been linked to ovarian failure as well as chromosomal instability (15, 22). Recently, novel mutations of this gene were also identified to associate with POI (23, 24). Large scale studies were previously conducted and they found that rs16991615 was associated with ANM in American populations with Hispanic, African and Indian descent, in addition to European women (17). This SNP was discovered to ramp up the risk of premature menopause (25). Bae et al. (26) and Day et al. (27) studies revealed association of rs16991615 with age at menopause at a genome-wide significance. The rs16991615 association with age at menopause was also replicated in the Iranian population (28). We identified that a minor allele of rs16991615 and TT genotype is not associated with increased risk of POI in our population. Similar to our results, Setti et al. (29) found no significant association between rs16991615 and poor ovarian response in Brazilian women. Moreover, other studies including Desai et al. (16) could not find any association between rs16991615 and POI. Recently, the minor allele of rs16991615 has been demonstrated to increase age-adjusted inverse anti-mullerian hormone (AMH), suggesting that factors affecting age at premature menopause could have a role in AMH regulation in ovarian reserves (30). For other SNPs including rs451417, we found that CC genotype was associated with an increased risk of POI by more than two-fold in comparison with careers of T allele.

We also investigated association of premature menopause with the rs244715, located in the *ZNF346* gene. *ZNF346* encodes a double-stranded RNA binding protein and takes part in apoptosis regulation (31). We confirmed that the minor allele of rs244715 could have an association with increased risk of the disease and MUAC; however, the study on Brazilian women failed to find any association between rs244715 and poor ovarian response (29).

Furthermore, Perry et al. (32) and Stolk et al. (14) found an association between rs1046089, located in the *PRRC2A* gene that encodes the HLA-B associated transcript, with age at menopause. Variations in this gene have been linked to different phenotypes, including BMI and height. Consistent with this, we noticed that the TT genotype of rs1046089 compared to C allele careers could be linked with increased risk of POI by more than 150%. However, Llácer et al. (19) found an increased risk of poor ovarian response in patients with CT and CC genotype, in comparison with TT genotype.

The rs7246479 and rs4806660 are located on chromosome 19 in the* TMEM150B* gene, which gives rise to transmembrane protein 150B, a member of TMEM150/Damage-Regulated Autophagy Modulator (DRAM) family. DRAM proteins play a role in apoptosis and autophagy (33). We found that women carrying GG genotype of rs7246479 were younger and had a lower rate of POI. Moreover, this SNP is also associated with age at menopause in the Chinese population (13). Furthermore, Setti et al. (29) found that rs4806660 has a protective effect against poor ovarian response. They further identified that the minor allele of rs4806660 was associated with enhanced controlled ovarian stimulation. However, the association was not statistically significant. Nonetheless, we demonstrated that C allele in the recessive model could associate with an increased risk of POI.

Another SNP associated with early menopause is rs2303369. The gene containing this variant encodes a protein called *FNDC4*. FNDC4 belong to the fibronectin type III domain family of proteins. FNDC4 binds to macrophages and monocytes. Administration of this could have therapeutic applications in inflammatory diseases (34). FNDC4 can also activate Wnt/β-catenin signaling pathway (35). Although Laisk-Podar et al. (36) established the association of rs2303369 with early follicular FSH, they could not find any association between rs2303369 and mean ovarian volume. On the other hand, we found that TT genotype of rs2303369 was associated with a significantly increased risk of POI in comparison with the major allele careers.

rs10183486 in the Tousled-like kinase 1 (*TLK1*) gene, encoding a serine/threonine kinase, is suggested to be involved in chromatin assembly, DNA repair and ovarian aging (37). Overexpression of *TLK1* in mouse embryonic stem cells has resulted in developmental arrest and apoptosis, as well as a decrease in pluripotency factors (38). We found that the minor allele of rs10183486 in comparison with the C allele and TT genotype in comparison with C allele careers (CT+CC) were associated with increased susceptibility to POI which is in concordance with the findings of Stolk et al. (14) study, reported an association between rs10183486 SNP and age at menopause.

Limitations of the current study include use of a population derived from a single region of Iran, hence it may not be possible to extrapolate our findings more widely. Secondly, we were unable to completely verify premature menopause by biochemical testing. Thirdly, the sample size of this study was quite small, which may affect the power of this study. Hence, there is a need to conduct further studies in different ethnicities with large sample sizes and to consider more comprehensive criteria for assessing the correlation between variants and premature menopause. 

## Conclusion

The rs16991615, rs244715, rs451417, rs1046089, rs7246479, rs4806660, rs10183486 and rs2303369 polymorphisms may potentially influence premature menopause. Although ethnic differences may result in diverse outcomes in association studies, more commonly recognized loci related to age at menopause could be subjected to replication in different populations. This could warrant further investigations with larger sample sizes to get a more comprehensive understanding of variations in different geographic regions. 

## Supplementary PDF


