# Supplementary material for: Genetic Determinants of Premature Menopause in A Mashhad Population Cohort
Source: Int J Fertil Steril. 2021 Jan 27;15(1):26–33. doi: 10.22074/IJFS.2020.134688 (PMC7838752; doi:10.22074/IJFS.2020.134688)
Supplement: Supplementary file 1 [file Int-J-Fertil-Steril-15-26-s01.pdf]

Int J Fertil Steril
International Journal of Fertility & Sterility
ISSN:2008-076X eISSN:2008-0778

www.ijfs.ir

Supplementary Information for

Genetic Determinants of Premature Menopause in A Mashhad Population Cohort

Mohammad Reza Mirinezhad, M.D.[1], Narges Khosroabadi, M.Sc.[2], Maliheh Rahpeyma, M.Sc.[1], Reza Khayami, M.Sc.[1, 3], Seyyed Reza Hashemi, M.Sc.[1, 3], Hamideh Ghazizadeh, Ph.D.[3, 4, 5], Gordon A Ferns, M.D., D.Sc.[6], Alireza Pasdar, M.D., Ph.D. [1, 7*], Majid Ghayour-Mobarhan, M.D., Ph.D.[4, 5*], Tayebeh Hamzehloei, Ph.D.[1*]

1. Department of Medical Genetics and Molecular Medicine, Faculty of Medicine, Mashhad University of Medical Sciences, Mashhad, Iran
2. Department of Genetics, Faculty of Biological Science, Shahid Beheshti University, Tehran, Iran
3. Student Research Committee, Mashhad University of Medical Sciences, Mashhad, Iran
4. Metabolic Syndrome Research Center, Mashhad University of Medical Sciences, Mashhad, Iran
5. International UNESCO Center for Health-Related Basic Sciences and Human Nutrition, Mashhad University of Medical Sciences, Mashhad, Iran
6. Brighton and Sussex Medical School, Division of Medical Education, Falmer, Brighton, Sussex BN1 9PH, UK
7. Division of Applied Medicine, Medical School, University of Aberdeen, Foresterhill, Aberdeen, AB25 2ZD, UK

Table 1: The primer sequences

SNP	Method	Primers (5´-3´)	
rs4806660	ASO	FI wild type	AGCTGCTTGCACATGCTTTACt
		FI mutant	AGCTGCTTGCACATGCTTTACc
		RO	ATGTTTACAGGCTAGGAGCAATG
		FO control	GAG AAG TCA ACG GCT AAG AGA C
rs451417	ASO	FI wild type	CGT TAT CAC CAT TGG GCT TCAA
		FI mutant	CGT TAT CAC CAT TGG GCT TCA C
		RO	GCT GAA TCT CAC ATA CCC TCT TC
		FO control	CCA TTG GTG AAG GGC ATA AAA CTA G
rs16991615	ASO	RI wild type	GAG TTG TAC CCT ACC TTC GTC
		RI mutant	GAG TTG TAC CCT ACC TTC GTT
		FO	GGC TAT CTT GAA TTG GAA ACT GC
		RO control	TCCCTACAATGACTCAACTAGA
rs7246479	ASO	FI wild type	GGCGGAGAAGTCAACGACG
		FI mutant	GGCGGAGAAGTCAACGACT
		RO	CTGGAGAGCATCGGAGACCA
		FO control	CAGTGGCTCATCTTGCTGGGTG
rs244715	ASO	RI wild type	AGTTTGGACAGAACCCCTTTACCACA
		RI mutant	AGTTTGGACAGAACCCCTTTACCACG
		FO	CAG GAC CAT TGA GAT AGT CAC AGGG
		RO control	TTATTAGGGTCTCACTCCGTTGC
rs1046089	ASO	FI wild type	TGGCACAGAACGATCACAGCG
		FI mutant	TGGCACAGAACGATCACAGCA
		RO	AGGCGTAAGTCTGAGTCCTGT
		FO control	GTCTCCTGCCTTCTTGTGATCACAGGAC

Received: July 5, 2020, Accepted: 7 September 2020
*Corresponding Addresses: P.O.Box: 8564-917791, Department of Medical Genetics and Molecular Medicine, Faculty of Medicine, Mashhad University of Medical Sciences, Mashhad, Iran
P.O.Box: 8564-917791, Metabolic Syndrome Research Center, Mashhad University of Medical Sciences, Mashhad, Iran
Emails: pasdara@mums.ac.ir, ghayourm@mums.ac.ir, hamzehloiet@mums.ac.ir

Int J Fertil Steril
International Journal of Fertility & Sterility
ISSN:2008-076X eISSN:2008-0778

Table 1: Continued

SNP	Method	Primers (5´-3´)	
rs780088	ASO	FI wild type	TTCAAGCAATTCTTCTGCCTCAGCCTACC
		RI	GCCTGTAACCCCAGCTACGCA
		FO	GCTCTCAACAGCCTCCTCTCCCTCTC
		RO	AGTGGCTCACGCCTGTAATCCCAGCACT
rs10183486	Tetra ARMS	FI wild type	GTT GGC TAA CAA CTT GAC TCA C
		FI mutant	GTTCAGATTCTTTGCCCAAGCA
		RO	GCA AAT CAG AAA TTC CCC
		FO control	ATTGCTGGATGTGAGCATCT

SNP; Single-nucleotide polymorphism, FI; Forward inner, RO; Reverse outer, FO; Forward outer, and RI; Reverse inner
